# Realist review of managed alcohol programmes for people experiencing alcohol dependence and homelessness: what works, for whom, and in what circumstances?

**DOI:** 10.1186/s12954-026-01416-y

**Published:** 2026-02-18

**Authors:** Hannah Carver, Emma King, Jessica Greenhalgh, Gillian W. Shorter, Bernie Pauly, Tessa Parkes

**Affiliations:** 1 Salvation Army Centre for Addiction Services and Research, University of Stirling, Stirling, FK9 4LA Scotland; 2 School of Psychology, Queen’s University Belfast, Belfast, BT9 7NN Northern Ireland; 3 TreAdd Research Group on Treatment and Addiction, Tampere University, Tampere, Finland; 4 Canadian Institute for Substance Use Research, University of Victoria, Victoria, BC V8P 5C2 Canada

**Keywords:** Realist review, Managed alcohol programmes, Homelessness, Alcohol dependence

## Abstract

**Introduction:**

People experiencing homelessness and alcohol dependence are vulnerable to a range of harms, and existing treatment options, which are often abstinence-based, are inadequate for this group because they may be unavailable, unsuitable, or not aligned with goals. Abstinence-based treatment programmes also rarely address underlying social and health issues faced by this population. Instead, alcohol harm reduction approaches provide individuals with support to reduce the harms associated with their drinking, without the need to stop drinking. Managed alcohol programmes (MAPs) are one harm reduction approach specifically designed for this group. MAPs provide alcohol in regulated doses through the day, alongside wider support for housing, physical and mental health, welfare, and social connections.

**Methods:**

A realist review was conducted to explore the current evidence base for MAPs. Realist reviews aim to synthesise existing evidence to examine the contexts, mechanisms, and outcomes of complex interventions, on the assumption that the outcomes of these interventions are directly caused by underlying mechanisms which have been activated in particular contexts. Twenty-four initial programme theories were developed and then tested using international evidence and refined to 11 programme theories.

**Results:**

A total of 60 sources were included in this review, highlighting a range of contexts, mechanisms, and outcomes relating to MAPs. The 11 programme theories demonstrate the need for MAPs in a context where abstinence-based treatment is the norm but is often unsuitable for this population. For MAPs to be successful for this population they need to enable autonomy, address clients’ needs, create a sense of hope and purpose, and provide access to healthcare and other activities. MAPs can lead to a range of positive outcomes for those who access them.

**Conclusions:**

Our theoretically informed exploration of service implementation can inform the design, development, and optimisation of future MAPs internationally. At a time when homelessness and alcohol deaths are increasing, innovative harm reduction approaches like MAPs are required to improve wellbeing and support health contextualised by the complex lives faced by some individuals.

## Background

People experiencing homelessness often use alcohol as a response to trauma and to cope with difficult life circumstances including poverty, lack of employment, and family conflict [1]. Alcohol use disorder (AUD) prevalence amongst adults experiencing homelessness has been found to be around 38%, and in some studies as high as 50%, around 10 times higher than the general population [2, 3]. Homelessness is a product of structural and systemic factors and people often experience cycles of homelessness prior to their substance use, impacting their housing [1, 4]. People who experience both homelessness and alcohol dependence are vulnerable to a range of harms such as alcohol poisoning and seizures, chronic health conditions, premature death, poor mental health, assault and injury, and social exclusion [5, 6].

Existing treatment options, which are normally abstinence-based, may be inadequate for those experiencing alcohol dependence and homelessness [7]. They can be difficult to comply with and may not effectively address underlying issues such as poverty and trauma [7]. Abstinence-based treatments are particularly inappropriate for those who are unwilling, or unable, to stop drinking [7]. Alcohol harm reduction approaches provide individuals with support to reduce the harms associated with alcohol use, without the need for them to stop drinking [4, 8]. Currently services based on harm reduction approaches are rare for those experiencing alcohol dependence and homelessness [4]. Managed alcohol programmes (MAPs) are one harm reduction approach that have been specifically designed for this group. MAPs provide alcohol in regulated doses through the day, alongside wider supports for housing, physical and mental health, welfare, and social connections [9]. MAPs were originally developed in Canada in the 1990s and have since extended across the world, including the United States (US), Portugal, Australia, the United Kingdom (UK), and Republic of Ireland. Other similar services are also likely to exist but might not label or promote themselves as MAPs so are hard to identify in mapping exercises. This is sometimes due to a desire to remain ‘below the radar’ due to social stigma directed towards both clients and services.

Evaluations of MAPs in Canada have found that they are associated with positive reductions in a range of areas including: alcohol consumption; withdrawal seizures; ambulance, emergency department and police contact; legal and social harms; and improvements in a wide number of factors including relationships and housing retention [10–15]. Previous systematic reviews have provided some evidence about the impact and effectiveness of MAPs [2, 16, 17]. A realist review provides a different approach, enabling an understanding of why MAPs work, for whom, and in what circumstances. A realist review is a “*method for studying complex interventions in response to the perceived limitations of conventional systematic review methodology. It involves identification of contexts*,* mechanisms and outcomes for individual programmes in order to explain differences*,* intended or unintended*,* between them*” [18]. Realist reviews aim to synthesise existing evidence to examine the contexts, mechanisms, and outcomes of complex interventions on the assumption that the outcomes of these interventions are directly caused by underlying mechanisms which have been activated in particular contexts [19, 20]. The aim of this realist review was to identify what works, for whom, and in what circumstances when delivering MAPs. By examining international evidence, we tested programme theories regarding the delivery of MAPs and synthesised data to provide a detailed understanding of the key components, mechanisms, and outcomes required for successful MAPs.

## Methods

We followed the five iterative steps of realist reviews [21, 22]: locate existing theories; search for evidence; select documents for inclusion in the review; extract and organise the data; and analyse and synthesise the data and draw conclusions.

### Step 1: locate existing theories

We developed initial programme theories (IPTs) of how MAPs operate internationally to understand what works, for whom, and in what circumstances. We examined the current evidence base through literature already known to the research team to identify the key components for MAPs [5, 9, 23–25]. Relevant theories were identified through the team’s expertise and using relevant theoretical frameworks. Twenty-four draft IPTs were developed and amended by the team before discussing with the study Learning Alliance, an advisory group of 15 people with relevant expertise including those with lived/living experience of alcohol dependence and homelessness, healthcare professionals, policy makers, homelessness services staff, and those delivering MAPs in other geographical areas such as England and Canada. Eight of these IPTs, those with most ambiguity, were selected for more detailed group discussion in an online meeting with group members where the study team provided an overview of each IPT and members were invited to share their views. These were then refined based on the feedback of the Learning Alliance, for example, clarity about the gaps that MAPs can fill (PT1), detail about mental health impacts (PT8), and information about increased healthcare costs (PT9). The final IPTs are shown in Supplementary File 1.

### Step 2: search for evidence

Searches were conducted to identify documents that could help us to further develop and refine our IPTs. The search strategy was developed by HC and EK, with input from the wider team and support from a University of Stirling specialist librarian. The search looked for all documents about MAPs from any date and any country and written in English. As we only wanted to capture papers on MAPs, the search strategy used the search terms ‘Manag* Alcohol Program*’ and was translated for use across different databases. Where possible, searches were limited to title and abstract as full text searches produced too many irrelevant hits. The acronym ‘MAP’ also produced too many irrelevant hits.

Searches were carried out by EK in March 2024 in CINHAL (via EBSCO), MEDLINE (via OVID), EMBASE (via OVID), HMIC (via OVID), APA PsycInfo (via EBSCO), Web of Science (via Clarivate), Health Source (via EBSCO), SocINDEX (via EBSCO), Cochrane, and SCOPUS. Google Scholar and Web of Science alerts were set up and ran for the duration of the review and results from these alerts were screened ‘on screen’. After screening, included studies from the main searches were subject to forwards and backwards citation chaining to identify any papers that might have been missed in the formal searches. Grey literature searches were conducted in April-June 2024 by EK. Several grey literature documents were already known to the team or supplied by members of our Learning Alliance. We also searched X, Google, Bielefeld Academic Search Engine (BASE), Overton database, and the Canadian Managed Alcohol Program Study website. Where Google searches highlighted relevant organisations, we also searched their websites for additional documents.

Titles and abstracts and then full texts were screened by EK and HC using Rayyan, with any conflicts discussed with GWS. Grey literature was screened by EK and HC and discussed, and documents identified via citation searching were screened by EK and GWS, with conflicts discussed with HC. Realist reviews are iterative and search terms and exclusion/inclusion criteria were open to be modified if necessary. Initial exclusion/inclusion criteria were deliberately broad to encompass all potentially relevant material. In the end we clarified the exclusion criteria to make clear that wet hostels (which allow alcohol but do not manage it) were excluded, as were programmes that manage alcohol through drugs/therapy but were not explicitly MAPs (see Table 1).

**Table 1:** Inclusion and exclusion criteria

Inclusion criteria	Exclusion criteria
All documents published in English, from any date and from any country. Type of intervention: Managed alcohol programmes (MAPs). Study design: all study designs. Types of settings: any setting providing managed alcohol programmes. Types of participants: all clients eligible for managed alcohol programmes. Outcome measures: all outcome measures related to managed alcohol programmes.	Harm reduction that did not include MAPs, for example wet hostels. References to programmes to manage alcohol that were abstinence based or using drug therapy only.

### Step 3: select documents for inclusion in the review

Steps 3 and 4 took place simultaneously. Full-text documents were divided equally between EK, HC, and JG. Full-text documents were selected for inclusion in the review by screening for whether the document can contribute to informing some aspect of the programme theory (relevance). At the point of inclusion documents were also assessed for how trustworthy and plausible the methods were to generate the data (rigour), although an assessment of rigour was not possible for all documents. A random sample of five files were coded by both EK and JG. Any disagreements were resolved by discussion between all three coders.

### Step 4: extract and organise the data

Data were extracted and organised by EK, HC, and JG. All sources were read in full and descriptive characteristics of the documents were entered into an Excel spreadsheet, for example details of authors, location, and type of MAP. The full texts were uploaded into NVivo and initially coded thematically using the IPTs as a framework. Memos were used to document thoughts about codes. This coding was deductive (to evaluate potential propositions included in the IPT), inductive (to enable new ideas and propositions to emerge from the evidence) and retroductive (to identify and explore patterns, using theory to offer causal explanations) [26, 27]. Due to the nature of the topic and papers it was difficult to extract individual context, mechanisms, and outcome configurations (CMOCs) into an Excel spreadsheet without further discussion. Instead, data were coded for the original IPTs, noting where data did not fit current IPTs, or where current IPTs did not encompass the data in the documents.

### Step 5: analyse and synthesise the data and draw conclusions

Analysis and synthesis were carried out to develop more refined realist programme theories (PTs), to explain what works, for whom, and in what circumstances with MAPs. EK, HC, and JG discussed the coding for each and redeveloped the original IPTs based on the evidence found. Some IPTs were truncated or removed, and new PTs were added. Supplementary File 2 provides information about these decisions. As realist review is an iterative method other documents were rescrutinised to search for any information relevant to the revised theories. Several PTs were also discussed with members of the study Learning Alliance and have been incorporated into the findings below. Propositions were developed to help explain underlying mechanisms, represented through CMOCs, to explain how context works to trigger mechanisms that provide different outcomes.

## Results

**Fig. 1:**
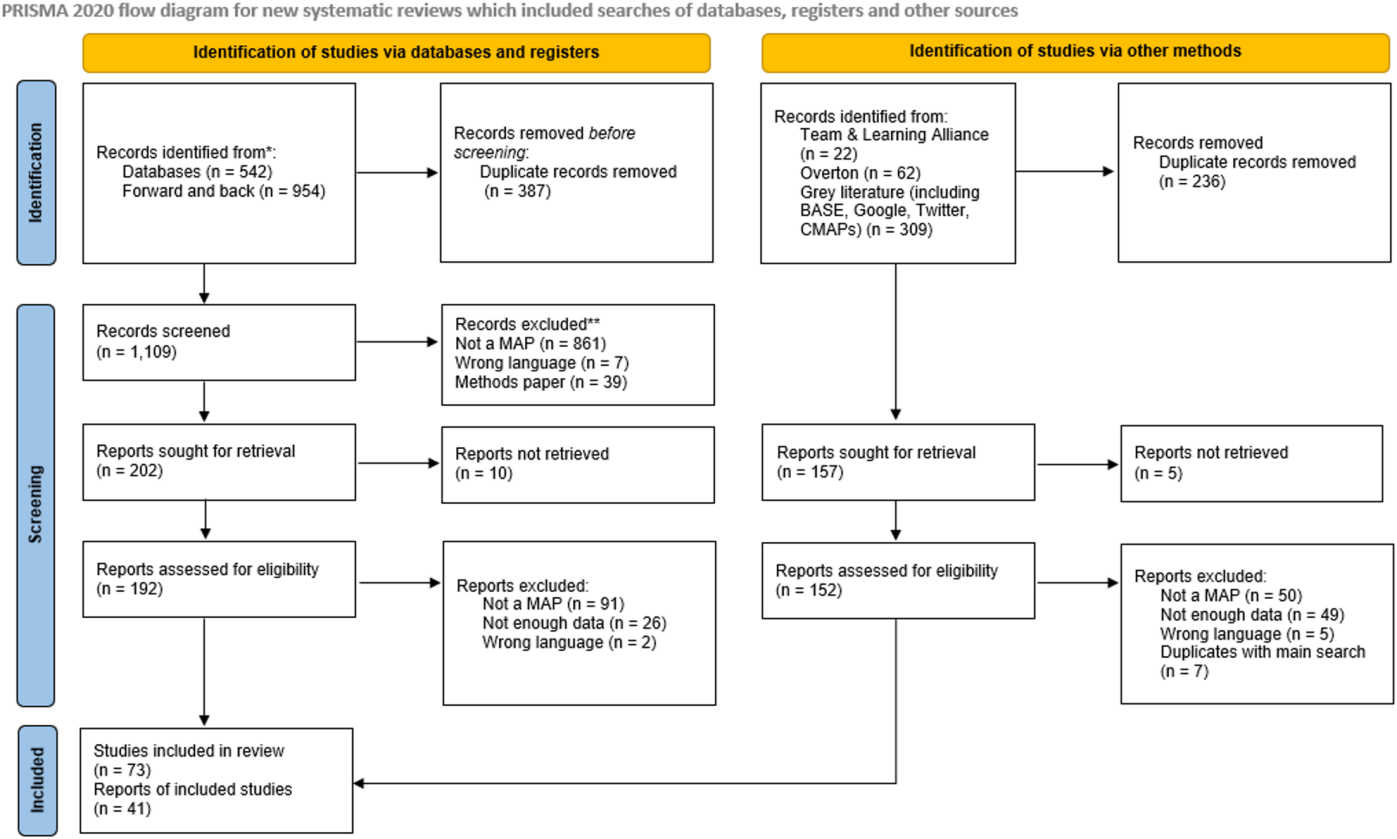
PRISMA flowchart

After removing duplicates, 1,109 records were identified via databases and registers. Of these 192 were screened at full text and 62 were eligible for inclusion in the review. In total, 163 grey literature sources were identified, 157 screened at full text, and 41 were included in the review. At the screening stage the main reasons for excluding papers were that they were not explicitly about MAPs or had too little information to be relevant (for example conference abstracts and documents in which MAPs were mentioned as an aside or in limited detail). We also made the decision to exclude papers solely focused on cannabis substitution in MAPs. In total, 114 documents were included at the review stage. After coding we selected 60 of these as core papers for the final review as they met the criteria of both relevance and rigour. Key characteristics of included papers were recorded (see Supplementary File 3) and the searches and screening results are shown as a PRISMA flowchart [28] in Fig. 1.

The final 11 PTs are detailed in Table 2 below. These PTs are written as CMOC statements, to highlight the causal relationship between contexts, mechanisms, and outcomes and are described in more detail throughout this section.

**Table 2:** Final programme theories

Programme theory (PT)
PT1: For people experiencing alcohol dependence and homelessness who are unwilling or unable to stop drinking (C), services are unsuitable (M) which means people cannot access the support they need (O) because of the expectation of abstinence (M).
PT2: MAPs (C) have high engagement (O) and offer those experiencing alcohol dependence and homelessness an option to obtain housing and help (e.g., food, training, medical care) (O) because they do not require them to give up alcohol (M).
PT3: MAPs (C) may be unsuitable and undesirable for some people (O) because they require clients to follow the rules set by the MAP (M) and must meet MAP eligibility criteria (M).
PT4: MAPs (C) allow alcohol to become a secondary part of people’s lives (O) and clients to be more engaged in other aspects of their lives (O) because they reduce the preoccupation of cravings and withdrawal, and stop clients searching for alcohol, by providing managed doses of alcohol at regular times (M) and provide opportunities for a range of activities (M).
PT5: MAPs (C) allow clients to make decisions about their lives (O) by giving them some choice, responsibility, and autonomy about their drinking and wider care (M).
PT6: MAPs (C) address clients’ needs (O) and allow them to develop trusting relationships (O) because MAPs are adequately resourced with suitable, well-trained and compassionate staff (M) and consider clients’ past experiences of trauma, stigma and poor continuity of care (M).
PT7: MAPs (C) reduce risky drinking (e.g., binging, non-beverage alcohol, unsafe settings) and the harms of alcohol (O) because they provide a safer physical environment (M).
PT8: MAPs (C) improve clients’ physical and mental health and quality of life, prevent them from deteriorating, and/or offer them a dignified death (O) because they provide regular clinical oversight (M) and support clients to engage with external healthcare services (M).
PT9: MAPs (C) can reduce interactions with emergency services (ambulance, police, A&E) (O) which results in cost savings (O) because they can reduce risky drinking behaviour, intoxication, and violence (M).
PT10: MAPs (C) support clients’ needs (O) because they operate at the intersection between existing services, local communities, physical place, and resources (M).
PT11: MAPs (C) create sense of hope and purpose (O) and improve quality of life (O) because they don’t stigmatise very marginalised people (M).

### PT1: For people experiencing alcohol dependence and homelessness who are unwilling or unable to stop drinking (C), services are unsuitable (M) which means people cannot access the support they need (O) because of the expectation of abstinence (M)

Alcohol policy in many countries, particularly North America, has traditionally followed a moderation or abstinence-based approach [4]. Gaetz (2018) argues that this is due to services and law enforcement adopting a “*strong moral perspective… that frames users as weak*,* a victim of bad choices*,* lacking judgement and/or courage*,* and so abstinence becomes the only solution*” [29]. For those experiencing alcohol dependence and homelessness, abstinence is not necessarily something everybody wants or is able to achieve presently [30–32] so this population are not having their needs met through this focus on abstinence-based services [33]. Where shelters and other services have required abstinence [4, 31, 32, 34–39], housing becomes contingent on abstinence and is based on the assumption that people comply with treatment for mental health and substance use problems [8]. Abstinence-based approaches have been criticised for a lack of understanding of the mechanisms behind behaviour change and a failure to respond to the additional needs of adults experiencing homelessness and alcohol dependence [31, 33, 35, 40]. For these individuals, being unable to access abstinence-based housing can result in violence, injury, and even death [12, 32, 36] because people may avoid shelters [3] or binge drink before attending in an effort to avoid withdrawal symptoms [39]. They can also face extreme alienation from services, leading to stigma and the potential dangers of drinking in public spaces, such as harassment [32, 41]. Those who have managed to maintain abstinence for a period of time report shame and embarrassment if they have gone back to drinking [12] or have been asked to leave housing due to their alcohol use [41–43].

Alternatives to abstinence-based treatment tend to focus on people with mild to moderate AUD, rather than those experiencing severe dependence and other vulnerabilities [4]. Similarly, strategies for harm reduction often focus on the whole population, such as pricing and regulation, which do not reduce harms in vulnerable individuals with AUDs and can cause additional harms such as binge drinking or non-beverage alcohol (NBA) use [2, 4, 44]. Community-based agencies were often the first to realise that this population were not adequately supported by traditional services [40]. MAPs arose from organisations who adopted models such as Housing First and operating wet shelters developing further approaches to address these gaps, recognising the need to provide fair access to housing, healthcare, and other services regardless of alcohol use [8, 35, 39, 41]:

*It is important to recognize that there are more than just abstinence-based ways to support people with severe alcohol problems*,* and MAPs provide a more compassionate response to people who have complex issues and run out of treatment and housing options.* [41]

Many of those who subsequently become MAP clients have had multiple previous interactions with services [45], often moving between emergency shelters, hospitals, and prison due to the lack of services available to them [12, 46–48]. These individuals have often been repeatedly denied help due to their drinking and are seen as less worthy of support, despite this population often requiring more help than their peers [3]. Consequently, there are few treatment options available for this ‘placeless’ population and without MAPs levels of unmet need in this population would likely be worse. However, the prevailing abstinence model has meant limited funding and resources have been attributed to non-abstinence approaches [49].

### PT2: MAPs (C) have high engagement (O) and offer those experiencing alcohol dependence and homelessness an option to obtain housing and help (e.g., food, training, medical care) (O) because they do not require them to give up alcohol (M)

MAPs offer a response to the needs of people experiencing homelessness and alcohol dependence who are unwilling or unable to become abstinent and cannot access abstinence-based services [29, 41, 44]. The provision of alcohol of known quality at regular intervals throughout the day [40, 50, 51] means MAPs can be a way of keeping people in services where they can receive additional support [39]. While not all MAPs provide housing, many offer consistent access to food and shelter, as well as managed alcohol [31, 35, 40–43, 47, 52, 53], through residential or supported housing based on harm reduction principles [35, 39, 52]. In doing so, MAPs offer support to those who have previously been excluded from services or viewed as ‘disengaged’ [12, 32, 42, 43, 53, 54]. MAPs can also reduce harm from NBA use [2, 39, 54, 55], either by providing regular beverage alcohol or allowing clients to ‘swap’ NBA for beverage alcohol [56].

There is a wide variation in how MAPs operate. Some provide food up to three times a day, with clients encouraged to engage in cooking and cleaning up, whilst in others individuals will buy their own food and cook for themselves [47]. Although most MAPs provide some form of housing, either on-site or in the community [41, 47], others operate as drop-ins which can be a good transition for those who may find a residential setting too restrictive [2, 42, 57]. Others report struggling in MAPs without accommodation as they often return to the streets at the end of the day, making them vulnerable to a broad range of harms, and NBA use [58]. Where housing is provided it is an important and valued aspect of the MAP [30, 53], addressing the harms of unstable housing, the street, or emergency shelters [12, 31, 53, 59]. Housing that is not contingent on abstinence has been shown to contribute to improved health outcomes and quality of life for this population [33, 52]. Although abstinence is not necessary, some MAP tenancies are contingent on clients still requiring or continuing to engage with the MAP [47].

Offering long-term accommodation is a key goal, allowing stability for clients and giving them time to work towards other goals. For some this may mean learning, or re-learning, life skills such as communication, self-care, and money management, as a transition to more independent living [39, 47, 52, 58–60]. It can also mean having the time and energy for (re)constructing identity and purpose [41]. Additionally, MAPs can support (re)building social connections with some MAP clients seeking to improve family relationships [39, 40, 53]. It is important for MAPs to create a sense of home that allows feelings of safety, stability, and belonging, with opportunities for daily activities and to relearn social skills [3, 12, 32, 40, 41, 43, 59, 60]:

*At baseline and subsequent data collection points*,* all of the participants consistently rated the quality of their housing very high in terms of safety*,* privacy*,* affordability*,* spaciousness*,* and friendliness. All of the participants remained housed during the course of the evaluation. None were evicted or became homeless. Thus*,* a major program objective*,* maintenance of housing*,* was achieved.* [61]

In line with Maslow’s hierarchy of needs, MAPs not only address physiological needs, but also attend to safety needs, love and belonging, and, in some cases, even esteem and self-actualisation [62]. There are, however, some concerns about the potential for people to become institutionalised [43] and some MAPs are reported to be more clinical, with restrictive rules that may make it difficult for people to maintain previous friendships whom were a supportive resource [56]. There is great variation in how MAPs are run: from the very low threshold Drinkers Lounge model in Vancouver, to Indigenous MAPs which reflect Indigenous approaches to wellness, to much more restrictive and clinical services where people have to be on site for a set time before they receive their next drink.

### PT3: MAPs (C) may be unsuitable and undesirable for some people (O) because they require clients to follow the rules set by the MAP (M) and must meet MAP eligibility criteria (M)

While MAPs may be the last treatment option available, they may still be unsuitable for some people. Review documents show that clients in MAPs were sometimes dissatisfied with the timings of alcohol administration and the strength and/or the amount of alcohol. In some MAPs they have no choice of the alcohol distributed, are not allowed to drink outside the MAP or bring drinks into the MAP [35, 38, 47]. For some clients who had been drinking at high levels for a long time, the amount of alcohol provided was deemed (by clients) to be very low, with clients finding this challenging and often resulting in people drinking outside the programme [3, 35, 37, 41]:

*“But a lot of others*,* they don’t feel that they get enough here” (R5004).*

*Another participant observed*,* “They are trying to get these guys to get in*,* not go out for drink. That’s why they have this program*,* but that is not enough for that guys because they are the alcoholic. It’s nothing” (R2014).* [35]

In order to limit outside drinking, some MAPs have clear policies, such as requiring clients to be on-site for up to 90 min before a serving, or not allowing any more alcohol that day if they leave the site [30, 41, 53]. When clients struggled to wait until their next alcohol serving, this could lead to hostility towards MAP staff [38]. Some MAPs also operate room searches and pat-downs to discourage clients from bringing alcohol back in [41]. In some, clients may be asked to leave if they are violent or repeatedly turn up under the influence [39, 59]. Some clients struggled with this controlled way of drinking [38] and others disengaged with the MAP because they wanted to live a ‘normal life’, to have their own home and relationships, and did not see this fitting with life in the MAP [36]. Understanding the type and extent of outside drinking in a MAP is crucial to reduce the risk of harm [63]. MAP clients may struggle with being in a shared accommodation space after many years on the streets and this can lead to arguments over issues such as cleaning [53]. Conversely, for MAPs where accommodation was not provided, transport could be a barrier to ongoing participation [58].

Clients must meet the eligibility criteria set by the individual MAP and be willing to join the programme [30, 33, 38, 53, 54, 59, 64]. Eligibility criteria are specific to each MAP but normally require the individual to be alcohol dependent and unstably housed, whilst other criteria might be police involvement, a history of harmful drinking, binge drinking and NBA use, frequent behavioural issues, and repeated unsuccessful attempts at abstinence [2, 8, 47]. Some MAPs are for men or women only or cater only to Indigenous men, and eligibility varies as to the levels of health and care needs an individual can have. Strict criteria and rules are mainly in place in an attempt to discourage outside drinking which could lead to clients drinking even more alcohol and creating increased acute and chronic health harms [41]. Staff are often positive about the need for strict eligibility criteria and clients had also asked for stricter criteria to make sure they could feel safe in their own homes [39, 61]. Clients may therefore require time to adjust to the rules and routines of the MAP [10].

### PT4: MAPs (C) allow alcohol to become a secondary part of people’s lives (O) and clients to be more engaged in other aspects of their lives (O) because they reduce the preoccupation of cravings and withdrawal, and stop clients searching for alcohol, by providing managed doses of alcohol at regular times (M) and provide opportunities for a range of activities (M)

Prior to joining the MAP, clients described an ongoing struggle to find money for alcohol, to obtain alcohol (or NBA), and to attempt to ration or store it to prevent withdrawal [12, 56]. For many this focus on alcohol was all-consuming [39]. The managed and reliable doses of alcohol provided by MAPs allowed clients to no longer be in ‘survival mode’, allowing them to think about other things [2, 33, 35, 60, 61, 65]. In Kidd et al.’s [36] case study of ‘Mark’ and his experience in a MAP, the authors noted:

*The MAP*,* as he described it*,* played a crucial role in Mark’s struggle to get back to a life and identity that held valued meaning for him. Through a harm reduction approach*,* free from the demands of street survival and not having to ‘‘crack off’’ his alcohol use*,* a space was provided for him to engage in this struggle in a way that had never before been possible in abstinence-based settings.* [36]

MAPs provide a range of other activities such as sports, arts and crafts, music, outside excursions, and cultural activities (e.g., for Indigenous clients), to help prevent boredom and disconnection [38, 41, 47, 51, 59]. Some MAPs also enable clients to take part in employment, for example within the MAP or through schemes set up by the local council. However, a downside of MAPs that required clients to be on-site for up to 90 min before their alcohol serving was that this restricted their ability to take part in activities outside the MAP [41].

### PT5: MAPs (C) allow clients to make decisions about their lives (O) by giving them some choice, responsibility, and autonomy about their drinking and wider care (M)

MAPs encourage clients to make their own decisions by meeting people where they are at [35], involving them in decisions, and giving them agency around their wider care [41, 66]. This might be as simple as clients having their own space where they will not be disturbed, something they did not experience on the streets or in temporary shelters [45]. Alcohol distribution in many MAPs is tailored to the needs of each participant [43, 55, 59–61], and services build in a degree of flexibility to adapt protocols to the needs of individuals [38, 40, 43]. These principles underpin MAPs and are a core part of harm reduction.

Although the primary focus of a MAP is to reduce harm, some clients may start to see a change in their relationship with alcohol and to have more autonomy over their own intake [35, 40, 41]. This may involve clients changing the type of alcohol they drink, trying to drink more slowly, having low- or no-alcohol beer or grape juice, having the occasional day without alcohol, or having the option to try detox and abstinence if they choose to do so [31, 36, 39, 45, 47, 50, 54, 55, 58]. Some MAP clients do, however, express concerns that it would be difficult to manage their own alcohol intake were they to move on from the service [2, 67], especially as many do drink outside their managed alcohol agreement [68].

MAP clients are encouraged to engage in decision making together and discuss suggestions for the running of the service and activities [2, 39, 47, 58, 60, 63]. They are also expected to engage with staff and assist with daily jobs around the MAP such as cleaning, meal planning, and tidying up after meals [12, 60]. Giving clients choice and autonomy can help to counteract previous experiences of stigma [12]. Binge drinking after being paid was a common problem for MAP clients and, alongside training on budgeting, some MAPs will retain or manage money for their clients [59, 60], something clients have reported as being helpful [53].

### PT6: MAPs (C) address clients’ needs (O) and allow them to develop trusting relationships (O) because MAPs are adequately resourced with suitable, well-trained and compassionate staff (M) and consider clients’ past experiences of trauma, stigma and poor continuity of care (M)

MAPs offer help and support to clients beyond the distribution of alcohol, with housing support, medical care, money management, counselling, life skills training, food, socialisation, and activities a key part of MAPs [3, 30, 35, 52]. MAPs can provide specialist support from trained staff such as nurses, doctors, community workers, and counsellors [40, 41, 49, 52, 54]. Care workers can support clients with day-to-day living, attend appointments, and assist with medication [41]. In a review of MAPs, all were found to have staff on-site 24 h a day, although these are not necessarily medical staff [41]. Some MAPs have 24 h or daily nurses, with occasional visits from doctors, and in others care workers support clients to access primary care in the community [33, 40, 41]. For MAP staff this often involves advocating for their clients to access services in an abstinence-based environment [12]. Some MAPs have clients who require personal care and may have trained staff such as health support workers on site [45]. Staff may also be called on to provide a safe environment by mediating relationships between MAP clients and family and friends on the outside [11, 53]. Clients spoke of the security of the MAP, knowing staff were on-site 24 h a day helped to build trusting relationships between staff and clients [32, 33, 35, 59]. Work specifically went into making MAPs feel like a secure home where both staff and clients felt valued and treated with dignity [45, 60, 66].

The care and compassion of the MAP staff was commented on in many of the review papers, especially for those who have been in and out of other services and experienced previous stigma [36, 38, 69]. As well as being compassionate and caring, MAP staff need to be well trained in order to deliver the flexibility required within MAPs [3, 43, 49]. Building trusting relationships between clients, staff, and healthcare providers is especially important for a population that has traditionally been marginalised [31, 36, 38, 41, 49]. For clients who have experienced previous trauma and stigma, this transition may take time [12, 31]. Staff were also aware that trusting relationships meant clients were more likely to be open with them about additional alcohol use, and they were mindful of how this relationship could potentially be damaged [38, 59].

Many clients have experienced hardships during childhood, intimate partner violence, other violence, poverty, and abandonment and therefore experience trauma, leading to negative thoughts, difficulties forming relationships, and self-destructive behaviours such as self-harm [38, 41, 43]. Drinking alcohol was identified by clients as a way of dealing with trauma [51, 53]. Staff often feel frustration at services, particularly those responsible for providing mental health support, that will not engage with those who are drinking, despite this being the main reason behind the alcohol use [59].

Particularly within a Canadian context, training staff on cultural awareness was important in many MAPs that provided services to Indigenous people, and such MAPs often bring in additional support from Elders, or for purification and sweat ceremonies [12, 38, 41]. It is important that staff understand the historical trauma of Indigenous communities which has led to high rates of substance use [33, 41].

MAPs often require an intensity of working with clients which requires more staff time per client to provide the required support [41, 61]. Needs of the clients could be diverse, leading to complex and often overwhelming workloads, in the context of pay lower than the population median which can result in high staff turnover [38]. Staff concerns were reported in terms of lone working or nighttime working with clients who may have been drinking outside their designated pours [59]. High staff turnover can lead to unease among clients who are just starting to form trusting relationships with staff and who may have already experienced poor continuity of care prior to the MAP which can lead to a team of staff who have not had much experience with MAP delivery [38, 50]. Staff would have also liked more time to take clients on outings outside the MAP [38].

### PT7: MAPs (C) reduce risky drinking (e.g., binging, non-beverage alcohol, unsafe settings) and the harms of alcohol (O) because they provide a safer physical environment (M)

MAPs provide a safe physical environment which is contrast to what clients had experienced in other shelters, on the streets, or in prison [11, 35]. This included feelings of security and stability, allowing them to sleep safely and have access to a warm place to stay, food, and healthcare [3, 4, 41, 50, 53, 59, 61].

MAPs encourage alcohol consumption in a less harmful way, through regular access to beverage alcohol, safe and clean sites rather than hiding their alcohol use, and without stigma, harassment, and physical violence found outside the MAP [4, 12, 31, 35, 37, 40, 41, 47, 58]. Access to regular alcohol reduces the need to beg, steal or consume NBA [13, 32, 40, 41, 50, 51, 65]. Some clients resorted to NBA use because they were excluded from establishments that serve beverage alcohol due to stigma and low income [33]. Clients who have just entered the MAP may continue to drink more alcohol, including NBA, but this gradually reduces compared to control participants (who are not in a MAP but who would meet the eligibility criteria) [13]. MAP clients may continue to drink more regularly than controls due to the availability of alcohol provision [13].

The consequence is that MAP clients experience fewer harms and a better quality of life compared to a similar population outside the MAP [2, 55, 66, 70, 71]. MAP clients have fewer interactions with criminal justice, and hospitalisations [39, 54]. There is also evidence that MAP clients have lower overall alcohol consumption, drink in a more consistent pattern, and are less likely to experience alcohol-related harms and poor liver function compared to controls [14].

### PT8: MAPs (C) improve clients’ physical and mental health and quality of life, prevent them from deteriorating, and/or offer them a dignified death (O) because they provide regular clinical oversight (M) and support clients to engage with external healthcare services (M)

MAP clients experience improvements in health and quality of life, and use fewer emergency care services, improve personal hygiene, experience few hospital bed days, drink less NBA and have a significantly decreased risk of death compared to controls [31–36, 39, 41, 44, 45, 46, 52–55, 59, 70, 71]. Prior to joining a MAP, clients often experienced a regular cycle of risky drinking and emergency hospital admissions [12, 58, 59], and they have been described as:

*… “frequent flyers”… “million-dollar men*,*” reflecting their intense use of emergency rooms*,* ambulances*,* prisons and other services that do not address their underlying problems.* [32]

Many clients experience improvements in physical health through increased eating, regularly taking their prescribed medication, and engaging with primary healthcare when in a MAP [39, 41, 43, 50, 52, 53, 58, 61]. MAPs can also help clients to attend appointments with podiatry, opticians, dentists, and sexual health services [59, 72]. Most experience fewer alcohol-related harms, such as blackouts and delirium tremens associated with withdrawal [43]. Clients also eat better, put on weight, and started to feel physically stronger [35]. There is also evidence that they are less likely to attend emergency departments and spend less time in hospital when in a MAP [73].

Many MAP clients experience severe and life-limiting illnesses prior to entering the MAP, as well as years of poverty and chronic alcohol use, and poor engagement with healthcare [54, 70]. While some improve, others may continue to show a decline in liver function in the MAP [39, 70, 71]. People experiencing homelessness requiring end of life care are often deterred from accessing services due to the requirement for abstinence [41]. MAPS can provide this end of life and palliative care to people who do not have their own family or are excluded from other services [40, 41]. MAPs must not just be seen as a place to die, however, with support for the majority of people to move on from the MAP when they are able to, for example into mainstream services or their own housing [42].

Much of the literature has focused on the impact of MAPs on clients’ physical health, with limited evidence of the benefits to their mental health [61, 64], although improved wellbeing has been reported [64]. Several papers reported collecting data about clients’ mental health but do not subsequently report it in the findings.

### PT9: MAPs (C) can reduce interactions with emergency services (ambulance, police, emergency departments) (O) which results in cost savings (O) because they can reduce risky drinking behaviour, intoxication, and violence (M)

Before entering a MAP many clients have regular interactions with hospitals, emergency services and criminal justice [12, 35]. These interactions have mostly been found to significantly reduce once in the MAP, sometimes by over 50% [31–33, 36, 40, 52, 54, 55, 59, 60, 71]. In MAPs the stabilisation of individuals’ alcohol use results in fewer alcohol-related harms, such as intoxication, disorderly behaviour, and injuries [31], where previously clients would cycle through binge drinking and emergency admissions [1, 12]. This represents a significant cost saving for the tax payer in providing emergency services [41, 44, 50, 74].

MAP clients reported an improved relationship with the police, with police services often happy to bring clients to the MAP rather than into custody [31, 41, 53]. MAP clients are encouraged to comply with criminal justice appointments [45].

Some interactions with health services, particularly primary care, increase for MAP clients as they are encouraged to seek healthcare more regularly [52, 53, 60]. This can lead to increased healthcare costs in some parts of the healthcare system, and reductions in costs associated with emergency care. Hospital-based MAPs have also been successful at encouraging patients to stay in hospital for treatment [71, 75], although concerns have been raised regarding the ethics of providing alcohol to inpatients, as well as the potential harms caused [76, 77].

### PT10: MAPs (C) support clients’ needs (O) because they operate at the intersection between existing services, local communities, physical place, and resources (M)

MAPs require local policies, created with clinical input, on how alcohol is administered to prevent health harms caused by daily administration [30, 44, 49]. Staff teams can fragment when individuals are in disagreement regarding harm reduction approaches, so appropriate training is required to overcome cultural stigmas of addiction [2, 49]. Because some staff identified a moral dilemma in supplying alcohol to people in a way that could cause harm, regular clinical oversight and clear guidelines were considered to be important [46, 49].

Some service providers considered the addition of a MAP to existing service provision as beyond the available resources and staff time [49]. It was highlighted that a lack of resources can be a barrier to ongoing support once a client moves to independent housing, and MAPs lack capacity for former clients to have short stay crisis admissions [60]. MAPs are generally funded through a mix of local and regional housing funds, special grants, or health systems, depending on the country/area [47]. Many MAPs rely on funding from multiple sources [47]. This leaves MAPs with challenges in securing ongoing funding, taking up staff time and leaving clients with instability of housing in MAPs that provide linked housing.

MAPs have been created with input from a variety of stakeholders, such as the local health department and local Business Improvement Areas, seeking to work out how best to provide help to their city’s most vulnerable populations [39]. In some countries, such as Canada, licensing laws mean that MAPs can brew their own alcohol on site [39], whereas in other countries, such as Scotland, individuals are responsible for purchasing their own alcohol [59].

MAPs generally do best when started by organisations that are already supporting this population and have a track record of delivering services [59]. It is also important that MAPs are connected to those existing services in the local community, such as counselling, substance use services, clinical support, and religious/Indigenous support, where necessary [13, 33, 54, 59, 61]. A harm reduction ethos is often not well understood by other local services, many of which instead operate as abstinence-based, with MAP staff having to navigate these policies to effectively support their clients [12]. Staff report having to advocate on behalf of their clients when accessing other services, particularly those services where abstinence is seen as the norm [12]. Consequently, education and training on harm reduction is an important aspect of MAPs [12, 49]. This also includes involving existing services in the planning of MAPs and advising services of the existence of the MAP, so that they are able to refer clients to it [59].

Space and place is important in a MAP, with review papers mentioning welcoming and trauma/psychologically informed environments that are safe and provide communal space for eating and activities [11, 59, 69] and in locations close to the communities in which they serve [30]. Beyond simply a place to be safe, many MAPs strive to feel like a home for their clients:

*Most of the participants highlighted that the MAP was not simply “housing” but a “home.” One resident stated*,* “you feel safe*,* you feel like you’ve got a warm place to stay*,* and you know*,* some home.” Many participants described the MAP residents and staff as being “like family.”* [11].

A MAP is foremost a residence and should appear more like a home rather than an institution [78]. Therefore, buildings should reflect this and should be designed to ensure they are welcoming rather than being too utilitarian and institutional.

### PT11: MAPs (C) create sense of hope and purpose (O) and improve quality of life (O) because they don’t stigmatise very marginalised people (M)

Many MAP clients report being stigmatised by services and other members of society before entering the MAP, for example for experiencing homelessness, street drinking, or drinking NBA [15]. Harm reduction as an overall ethos seeks to provide people with help and support, regardless of their current circumstances. MAPs are seen by staff and clients as providing a non-judgemental service which tries to overcome the sense of shame and stigma that has often been internalised by clients after years of stigmatising treatment. Pauly and colleagues (2013) note:

*Both residents and staff indicated that the program was different from other programs because of the non-judgmental approach to substance use. For example a staff person stated*,* “I think the program is just about providing housing that is completely non-judgmental.”* [53].

MAPs can also increase clients’ sense of hope and purpose. Clients in several studies talked about the MAP as giving them a sense of hope for the future, with goals around housing and employment [11, 12, 53, 58]. As Pauly et al. (2019) note, this sense of hope is “*in direct contrast to the pre-MAP arena where their needs were largely unmet with little hope for the future*” [12]. Improved liver function [10] and services staffed by those with lived experience [24] were two examples of factors increasing hope. MAPs can also give individuals a purpose in life. Participants in a study by Motta-Ochoa et al. (2025) reported a loss of purpose and boredom as a result of the regular provision of alcohol [38]. Ensuring MAP clients are provided with a range of meaningful activities is key to enhancing their sense of purpose and reducing boredom [36, 38, 59].

MAPs also allow clients to reconnect with families and friends, and to develop and maintain positive relationships [53, 61], which many positively compared to the unhealthy relationships they had experienced prior to entering the MAP [59]. The MAP is an accepting environment for those who have previously been excluded by family and communities [54]. Clients learned to develop positive social interactions within the MAP and build a sense of community, peer support, and a feeling of security and safety in a supportive environment [54, 59].

## Discussion

This realist review has allowed a theoretical understanding of the ways in which MAPs work, for whom, and in what circumstances. In this paper, we have described 11 refined programme theories, and the contexts, mechanisms, and outcomes within each one. By developing these programme theories, there can be a deeper understanding of what is required for MAPs to operate successfully.

Our findings show that MAPs are required in contexts where alternative services are unsuitable and do not meet people’s needs, often due to the expectation of abstinence [4].

MAPs were developed because of the lack of harm reduction approaches for those experiencing homelessness and alcohol dependence, with an increase in demand for MAPs in response to the COVID-19 pandemic [79]. When housing services expect abstinence, individuals may choose not to engage or consume their alcohol in an unsafe manner prior to entering the services. Because MAPs operate within a harm reduction ethos, no expectations are placed on individuals stopping drinking; instead, their alcohol is managed to reduce harms including withdrawals. The provision of housing and other support in MAPs is viewed as beneficial in reducing the harms associated with risky drinking behaviours. While housing is not provided in all MAPs, when it is provided, clients view it as an important element of the service [30, 53].

Along with regular alcohol provision, MAPs can also provide food, activities, and feelings of safety and stability [47]. These findings of trust, safety, and inclusion are mirrored in other realist reviews of harm reduction interventions (e.g., drug consumption rooms [80, 81]) illustrating the importance of options including and beyond abstinence-based approaches. The regular provision of alcohol within MAPs can enable people to engage in other activities, including employment, because they are no longer preoccupied with finding their next drink [2, 33, 35, 60, 61, 65]. Individuals can make decisions about their lives, their alcohol use, and wider care as part of the autonomy facilitated by MAPs [41, 66].

MAPs can also reduce interactions with emergency healthcare and police/criminal justice because people are often not practicing risky drinking behaviours [31, 32, 36, 40, 52, 60, 71]. Such reductions in emergency service use can have cost savings [41, 44, 50, 74]. They can also reduce individuals’ likelihood of risky drinking which results in fewer harms and a better quality of life. Individuals are encouraged and supported to access mainstream healthcare which can lead to improved physical and mental health and quality of life [52, 53, 60]. MAPs can create a sense of hope and purpose due to the non-stigmatising and non-judgemental way in which they work with people [11, 12, 53, 58].

While MAPs are beneficial for many, they may be unsuitable for some because of the rules and eligibility criteria required [35, 38, 47]. Some may find the timings of alcohol administration, a limited choice of drink strength or amount, and rules around drinking outside particularly problematic, with some MAPs being stricter than others, and with varying degrees to which individual MAP clients are consulted in their alcohol administration plans [3, 35, 37, 38, 41]. While clear eligibility criteria are required, this may restrict who can access the MAP, especially those which are only for men or Indigenous individuals [2, 8, 47]. The staffing of MAPs is important, with well-trained and compassionate staff, and trauma-informed care enabling clients to build trusting relationships, something that may not have been possible in other services [36, 38, 69]. Regular clinical oversight, clear alcohol management guidelines, and staff training are essential. Well delivered, welcoming services, in locations that are both connected to local services and close to the communities that they serve, are likely to be effective [11, 30, 59, 69].

### Strengths and limitations

There are several strengths of this review. Firstly, our programme theories were developed and rigorously tested through empirical research, with input from our established Learning Alliance group. Using a realist review approach enables a comprehensive understanding the impact of MAPs on those using these services. While a traditional systematic review would have provided insight into the evidence base, this realist review goes further by providing an understanding of not only the key components of MAPs but the behavioural mechanisms and context that can influence these. Inclusion of academic and grey literature sources from several countries is another strength. A limitation is that most of the literature included in the review was from Canada where the evidence base is most robust which may impact transferability to other countries. Additionally, while we specifically excluded literature about wet hostels/services as these are different to MAPs, we did find some articles that included MAPs under the umbrella term of wet hostels. This resulted in screening more articles in full text to include all references to MAPs. A small number of papers discussing MAPs using the term ‘wet hostel’ are therefore likely to have been excluded. Finally, it is important to acknowledge the limitations of realist review methodology: while the programme theories aim to explain causation, they may be incomplete or imperfect [20]. Future research, therefore, should test these programme theories empirically to further refine them. The findings could be viewed as subjective; to mitigate this we registered our protocol with PROSPERO to enhance transparency followed the robust realist review quality standards [82], and several team members were involved in each stage of the review.

## Conclusions

For those experiencing alcohol dependence and homelessness, abstinence-based services are often unsuitable. MAPs take a harm reduction approach and were specifically developed for this group in order to provide a safer alternative to traditional services. This realist review aimed to understand what works, for whom, and in what circumstances for MAPs. The 11 programme theories demonstrate the need for MAPs in a context where abstinence-based treatment is the norm but is often unsuitable for this population. For MAPs to be successful for this population they need to enable autonomy, address clients’ needs, create a sense of hope and purpose, and provide access to healthcare and other activities. MAPs can lead to a range of positive outcomes for those who access them. The findings of this review can inform policy and practice, contributing to the development and implementation of MAPs internationally. The review highlights many beneficial factors that can inform the development and delivery of MAPs to ensure they are successful for those using them. At a time when homelessness and alcohol deaths are increasing, innovative harm reduction approaches like MAPs are required.

## Data Availability

All data and materials included in this review are publicly available.

## Abbreviations

AUD: Alcohol use disorders
CMOCs: Context, mechanisms, and outcome configurations
IPTs: Initial programme theories
MAP(s): Managed alcohol programme(s)
NBA: Non-beverage alcohol
PRISMA: Preferred reporting items for systematic reviews and meta-analyses
PTs: Programme theories
US: United States
UK: United Kingdom
